# Isolation and characterization of bovine alphaherpesvirus 2 strain from an outbreak of bovine herpetic mammillitis in a dairy farm

**DOI:** 10.1186/s12917-020-02325-3

**Published:** 2020-03-30

**Authors:** Gianvito Lanave, Vittorio Larocca, Michele Losurdo, Cristiana Catella, Paolo Capozza, Maria Tempesta, Vito Martella, Canio Buonavoglia, Michele Camero

**Affiliations:** Department of Veterinary Medicine, University of Aldo Moro of Bari, 70010 Valenzano, Italy

**Keywords:** Bovine, Alphaherpesvirus, Mammillitis, Bovine alphaherpesvirus 2, BHM

## Abstract

**Background:**

Bovine alphaherpesvirus type 2 (BoHV-2) belongs to family Herpesviridae, subfamily *Alphaherpesviridae* and can cause two distinct, well-defined conditions: a generalized benign skin infection that somewhat mimics lumpy skin disease (LSD), referred to as Pseudo-Lumpy Skin Disease (PSLD) and a localized ulcerative mammillitis, referred to as Bovine Herpetic Mammillitis (BHM). BHM is a localized form of BoHV-2 infection that causes erosive-ulcerative self-limiting lesions on breast and nipples. BHM is chiefly a disease of lactating dairy cows and has been described sporadically in several countries. In this study we describe an outbreak of bovine herpetic mammillitis caused by BoHV-2 occurred in a dairy farm in Southern Italy. Clinical signs were observed in 26/59 lactating cows with the age ranging between 2 and 6 years. The affected animals were afebrile, showed lesions on the skin of nipples, breast and ventral surface of the abdomen, near the mammary veins and spontaneously recovered within 2 months.

**Results:**

BoHV-2 DNA was detected in the crust samples by pan-herpes PCR and real-time quantitative PCR. The virus was isolated on bovine kidney cells and was characterised by deep sequencing technologies. The nucleotide identity to BoHV-2 of the strain ITA/2018/468 retrieved in this study ranged from 98.83 to 100%. Phylogenetic analyses based on three full-length gene (glycoprotein B, thymidine kinase and glycoprotein G) sequences confirmed the close relatedness of the strain ITA/2018/468 to BoHV-2 sequences.

**Conclusions:**

The report represents a significant outbreak of BHM in a dairy farm 50 years after the last description in Italy. However, outbreaks of PLSD have been described in Europe recently, indicating that the virus is present in European territories. Improving the diagnostic algorithms and enacting specific surveillance plans could be useful to understand better the epidemiological and pathogenetic patterns of BoHV-2 infection in livestock animals, and to develop, eventually, effective prophylaxis plans.

## Background

Several herpesviruses (family *Herpesviridae)* of the subfamilies *Alphaherpesviridae* and *Gammaherpesviridae* are able to infect cattle, with bovine alphaherpesvirus (BoHV) -1 being regarded as a major bovine pathogen [1].

BoHV-2, an alphaherpesvirus antigenically related to human herpes simplex virus 1, was first isolated in South Africa from generalized skin infections of cattle and it was named Allerton virus [2]. BoHV-2 can cause two distinct, well-defined conditions: a generalized benign skin infection that somewhat mimics lumpy skin disease (LSD), referred to as Pseudo-Lumpy Skin Disease (PSLD) [3] and a localized ulcerative mammillitis, referred to as Bovine Herpetic Mammillitis (BHM) [4].

PLSD is clinically indistinguishable from LSD, a disease of cattle caused by a poxvirus and subjected to mandatory reporting. PLSD-affected animals show cutaneous nodular lesions of variable diameter, scattered throughout the body. The nodular lesions develop a central depression that heal without formation of scars and do not produce the deep necrotic sequestra typical of LSD [1, 3, 5, 6]. PSLD is widespread in Southern Africa, with sporadic reports from Australia, United Kingdom, United States, and Israel [1].

BHM is a localized form of BoHV-2 infection that causes erosive-ulcerative self-limiting lesions on breast and nipples [7, 8]. BHM is chiefly a disease of lactating dairy cows but also occurs in heifers about to calve and in beef animals [1]. BHM has been described sporadically in the United States, Canada, Great Britain, Europe, Africa, and Australia [1]. Detection of antibodies to BoHV-2 or the detection of viral DNA in several species of asymptomatic wild animals has been reported in Africa, Asia, and Hungary, thus suggesting that the infection is much more common than the disease [1].

The natural mode of transmission of BoHV-2 is not clear but it is presumed to involve mechanical vectors, particularly the milking machine, or the dermal inoculation by biting flies, such as *Stomoxys calcitrans* [1, 9]. Intact teat skin is refractory to virus penetration, indicating that some form of teat trauma precedes infection [1]. Herewith we report the molecular detection, isolation and characterization of a BoHV-2 strain from an outbreak of BHM in a dairy farm in Italy.

## Results

### Viral isolation

**Fig. 1:**
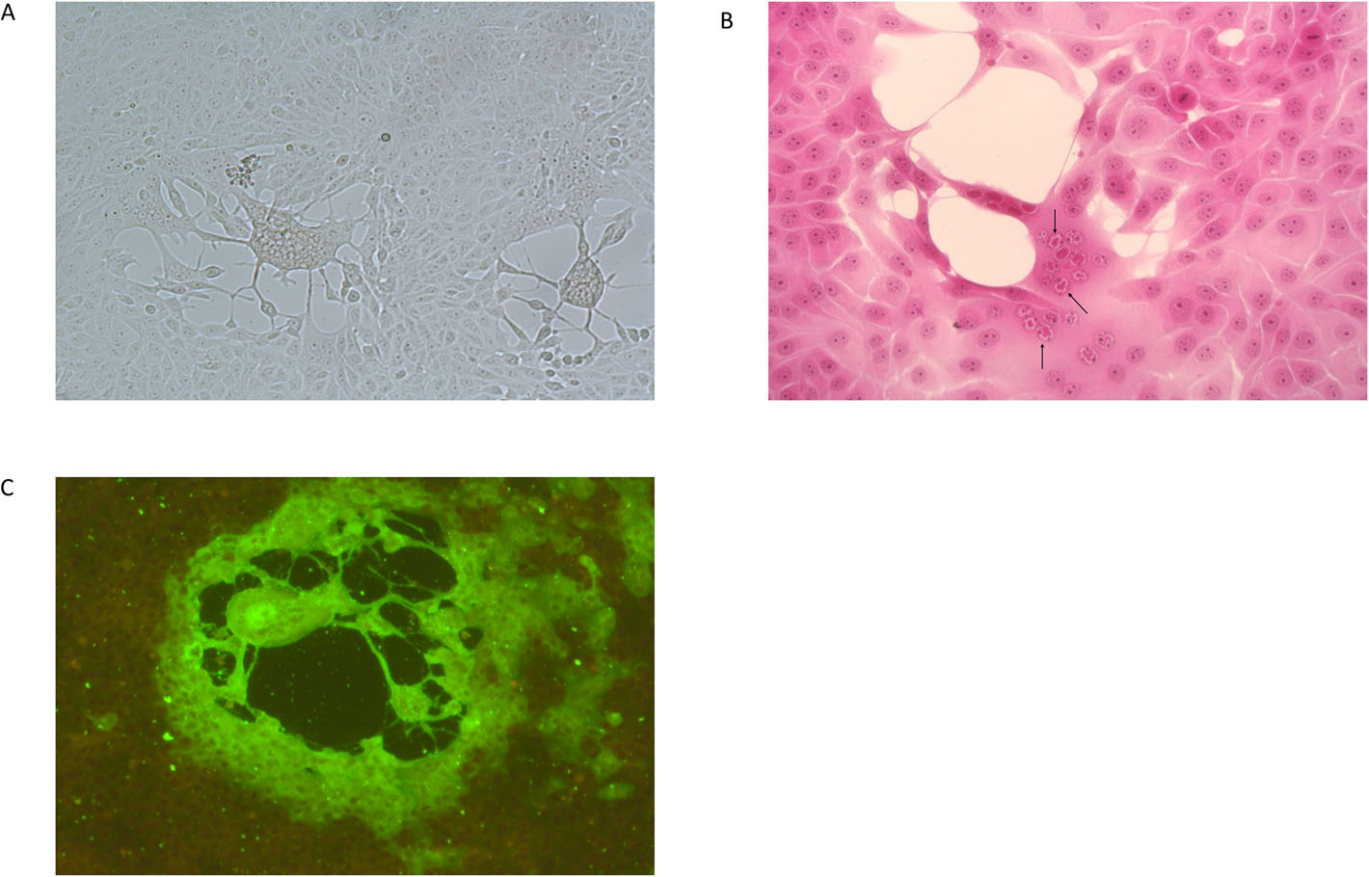
MDBK cells monolayer infected with BoHV-2 strain isolated from skin lesion from cows. Evidence of cytopathic effect in unstained infected cell monolayer (**a**). Nuclear inclusion bodies within syncytia are highlighted by black arrows (haematoxylin and eosin stain, original magnification × 400) (**b**). Nuclear fluorescence (original magnification × 400) (**c**)

Twenty-six skin samples collected from the lesions were processed for viral isolation on Madin Darby Bovine Kidney (MDBK). The inoculated cells showed cytopathic effect (CPE) within 96 h after infection, with rounding of cells, increased granularity and cell detachment from the monolayer (Fig. 1a). The extent of CPE varied with the samples, from isolated foci to extensive damaging of the cell monolayers. In the cells stained with haematoxylin and eosin large eosinophilic nuclear inclusion bodies were visible (Cowdry A), that were consistent with herpesvirus replication (Fig. 1b).

Upon immunofluorescence (IF) test, the infected MDBK cells showed granular fluorescence areas with the presence of the organised inclusions (inclusion bodies) in the nucleus (Fig. 1c).

### Screening with pan-herpesvirus PCR

A total of 20 samples tested positive for BoHV-2 DNA in first-round PCR and 23 in second-round pan-herpesvirus PCR [10].

Upon sequence analysis of the 222 nt-long amplicons generated in second-round PCR, the samples revealed high nucleotide (nt) identity (98.1–98.9%) to the DNA polymerase of BoHV-2 strain BMV (GenBank accession no. AF181249).

### Analysis with real-time quantitative PCR

The qPCR assay detected a total of 1.30 × 10^5^ viral DNA copies/10 μL in skin sample homogenates of the strain ITA/2018/468 and 5.56 × 10^5^ viral DNA copies/10 μL in the second passage onto MDBK cells. The qPCR assay was also used to re-screen the crusts of the animals. BoHV-2 DNA was detected by qPCR in 24 out of 26 samples, with viral load ranging from 2.50 × 10^2^ to 1.30 × 10^5^ viral DNA copies/10 μL. The sensitivity of detection of the assay was > 10^1^ viral DNA copies/10 μL of standard DNA and 3.86 × 10^1^ viral DNA copies/10 μL of DNA template, respectively. BoHV-2 quantification had an acceptable level of repeatability over various magnitudes of target DNA concentrations, when calculating the intra-assay and inter-assay coefficients of variation within and between runs, respectively [11]. Moreover, the qPCR did not recognise the DNA of bovine alphaherpesviruses (BoHV)-1 and BoHV-4 and bubaline alphaherpesvirus (BuHV)-1, suggesting a good specificity.

### Identification of BoHV-2 by next generation sequencing (NGS)

A total of 226,376 paired reads with a quality score > 99% were obtained from NGS. The reads were mapped to 23 different gene targets available in GenBank database and used as reference: the partial gene of terminase and the complete genes of the tegument protein, capsid protein, major capsid protein, integral membrane protein, glycoprotein H, thymidine kinase, non-glycosylated membrane-associated protein, capsid associated tegument, capsid maturation protease, glycoprotein B, nuclear phosphoprotein, DNA-dependent DNA polymerase, ribonucleotide reductase protein, nuclear protein, virion host shutoff protein, DNA polymerase processivity factor, membrane protein, glycoprotein C, tegument envelope protein, helicase primase, nuclear matrix-associated protein and glycoprotein G.

Upon comparison of the gene targets of strain ITA/2018/468 with cognate BoHV-2 gene sequences revealed a range of nt identity of 98.83 to 100% (Supplementary Table 1).

The sequences of three full-length gene targets (glycoprotein B, thymidine kinase and glycoprotein G) were available for all members of the genus *Simplexvirus* officially recognized by ICTV. Phylogenetic analyses were carried out using Bayesian inference. Posterior probabilities percentages were consistently high (> 95%) for all clades of the phylograms, supporting the inferred phylogenetic relationships.

**Fig. 2:**
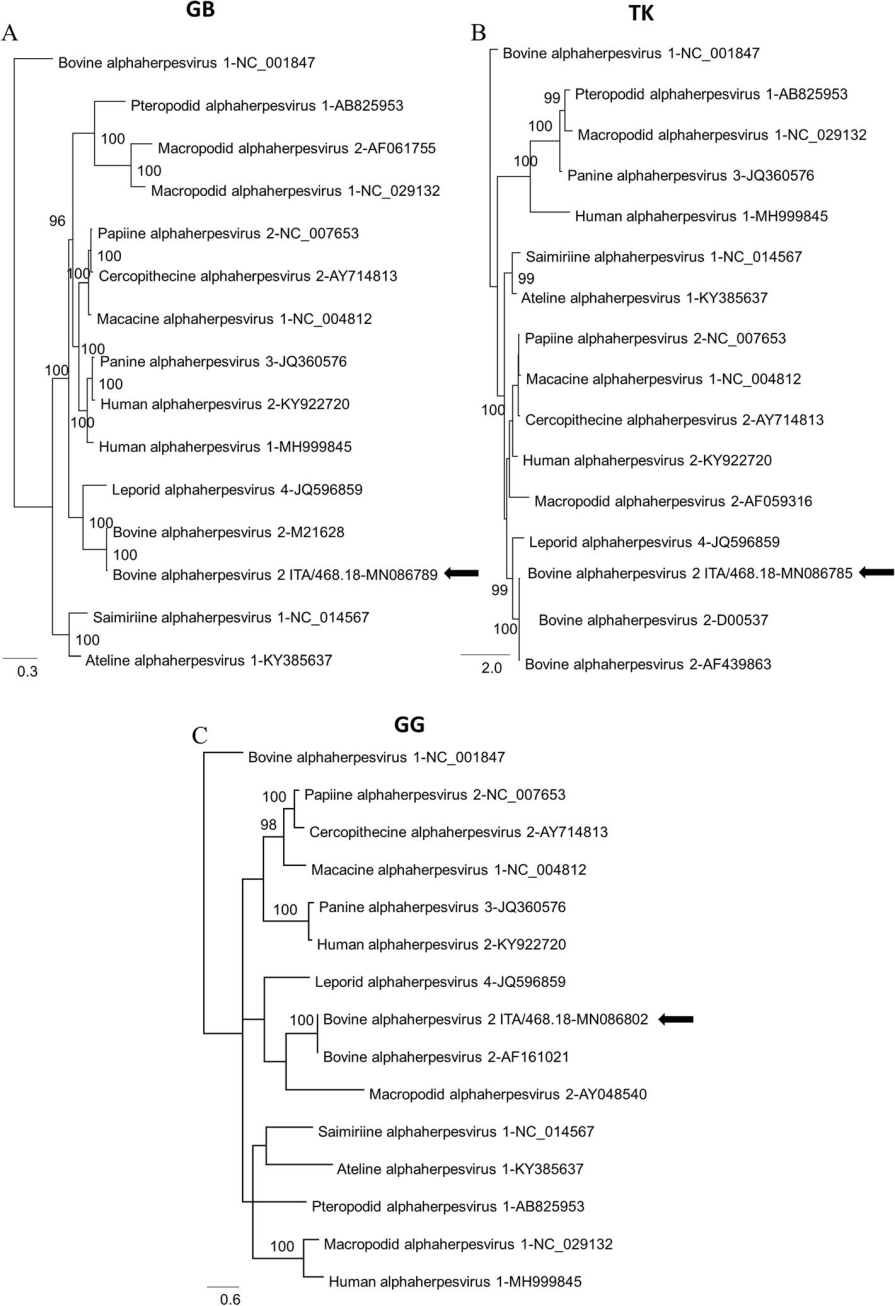
Phylogenetic trees based on the full-length nucleotide sequence of different target genes: glycoprotein B (**a**), thymidine kinase (**b**) and glycoprotein G (**c**) from the BoHV-2 ITA/2018/468 strain isolated in the present study or retrieved from International Committee on Taxonomy of Viruses (ICTV) database. Posterior output of the tree was derived from Bayesian inference using 4 chains run for > 1 million generations, a general time-reversible model, a proportion of invariable sites, a gamma distribution of rate variation across sites, and a subsampling frequency of 1000. Posterior probability values > 95% are indicated on the tree nodes. The black arrows indicate the BoHV-2 ITA/2018/468 strain isolated in this study. BoHV-1 (Genus *Varicellovirus*) strain (NC_001847) was used as an outgroup. Scale bars indicate nucleotide substitutions per site. GB, glycoprotein B; TK, thymidine kinase; GG, glycoprotein G

In the consensus phylogenetic trees (Fig. 2) based on the glycoprotein B, thymidine kinase and glycoprotein G gene sequences, strain ITA/2018/468 was closely related to other BoHV-2 sequences and grouped with either Leporid alphaherpesvirus 4 (LepHV-4) (JQ596859) (Fig. 2a and b) or with Macropodid alphaherpesvirus 2 (MacHV-2) (AY048540) (Fig. 2c) in the phylogenetic trees. Strain ITA/2018/468 displayed 57.2 to 75.3% nt identity in the three genes to LepHV-4 and 52.2 to 64.1%. to MacHV-2.

### Serological investigations

Serum samples were collected from 26 animals. Five bovine sera displayed antibodies specific for BoHV-2 with titers of 1:2 at T0, T15 and T45. Twenty-one sera did not display antibodies specific for BoHV-2 at T0, whilst the titers increased to 1:4–1:8 at T15 and to 1:8–1:64 at T45. Ten sera were collected from a different group of animals from the same herd that were clinically healthy. Eight of 10 sera tested negative for BoHV-2 at T0, T15 and T45, whilst 2 sera showed BoHV-2-specific antibodies titers of 1:4 at T0, T15 and T45.

## Discussion

BoHV-2 causes primary cutaneous infections in cattle and is common worldwide [12–14]. The virus is responsible for two different clinical forms of skin disease, namely BHM (localized) and PLSD (systemic), although both in natural and experimental infections sometimes a clear distinction between the two forms is not possible [15]. BHM causes serious economic losses and occurs more commonly in lactating dairy cows, and less frequently in beef cows, pregnant heifers and suckling calves [1].

We report the detection of a BoHV-2 strain, ITA/2018/468, from a BHM outbreak in a bovine dairy herd in Apulia region, Southern Italy. The outbreak spontaneously resolved 2 months after the onset of the clinical signs. Herpesvirus DNA was identified in skin samples by PCR using broadly reactive primer sets, able to amplify herpesviruses and the virus was characterised as BoHV-2 by direct sequencing of the amplicons. A quantitative PCR assay, more specific and sensitive, was also used to detect and quantify viral load. An isolate was obtained from skin samples and used for detailed characterization using NGS protocols. Analysis of 23 gene targets of the virus revealed a high nt identity (98.83 to 100%) to BoHV-2 reference strains. Upon phylogenetic analyses based on three different full-length gene targets (glycoprotein B, thymidine kinase and glycoprotein G) available for all the official members of the genus *Simplexvirus*, the virus appeared strictly related to BoHV-2 and grouped with either LepHV-4 or with MacHV-2 in the phylogenetic trees.

The first report of BoHV-2 in Italy dates back to the early 1970s [12]. In the 1972 study, an isolate, strain 69/1 LO, was made from a calf with gingival ulcerative lesions, in Umbria, Central Italy. Since then, for nearly 50 years BoHV-2 has never been reported in Italy. The reasons for this can be several. For instance, the disease is not included in the national mandatory prophylaxes plans for livestock animals. Also, since BoHV-2 infection in the herd is self-limiting, BHM is often tolerated/accepted by the farmers. However, the multifocal BoHV-2-related disease, PLSD, requires differential diagnosis with the poxvirus-related form (LSD), a zoonosis subjected to mandatory reporting in European countries. Interestingly, a recent study [16] has documented a PLSD outbreak in a small Piemontese cattle breeding in Northern Italy. The animals presented multifocal papular lesions evolving into persistent areas of alopecia. This confirms the hypothesis that the infection is not uncommon in Italian livestock animals whilst estimates of its actual prevalence are not available. In most cases, also, the transmission modes of the infection remain unknown.

In our case, upon reconstruction of the herd history, the BHM outbreak appeared related to the introduction of new animals in the herd. Fifteen alpine grey cows had been imported from Bolzano prefecture, Trentino Alto Adige, Northern Italy, about 1 year before the onset of BHM in the herd. A possible explanation for the disease outbreak could be the reactivation of the virus from a latently infected animal. Latent infections usually are reactivated by stress and are regarded as the most feasible source for the spread of the infection within a herd. Latency is characteristic for herpesviral diseases and reactivation of experimental BoHV-2 infection after treatment with corticosteroids has been demonstrated [17]. Although BoHV-2 DNA has been identified in trigeminal ganglia of cattle [18], the site of latency has not been identified. Calving could represent a reactivating stress factor and this might explain why cows, which have recently calved, are often severely affected by the disease [19]. Overall, interpretation of serology results in herpesvirus infections may be difficult. Although some herpesviruses can persist life-long in their host in latent infections [20], herpesvirus-specific antibodies can fluctuate markedly and eventually disappear temporarily or remain at very low titers [21]. Also, antigenic cross-reaction among genetically-related herpesviruses can confound the serological data [22]. In our case, sero-conversion was observed clearly in 21 animals with BHM, with a marked increase in the antibody titers from T0 to T45. In 5 animals with BHM, however, this pattern was not clear. Also, specific antibodies, at low titers, were detected in animals without clinical signs from the same herd.

## Conclusion

In our study, the outbreak affected 26 out of 59 lactating cows, in a herd with an overall population of 120 animals. This epidemiological pattern is consistent with the presence of immunologically naïve animals being exposed almost simultaneously to a common virus source. The spread of BHM exclusively to lactating cows could be related to the milking machine. Improving the diagnostic algorithms and enacting specific surveillance plans could be useful to understand better the epidemiological and pathogenetic patterns of BoHV-2 infection in livestock animals, and to develop, eventually, effective prophylaxis plans.

## Methods

### Outbreak description

In September 2018 an outbreak of BHM occurred in a bovine dairy herd located in Cassano Murge, prefecture of Bari, Apulia, Italy. The herd consisted of 120 animals (59 lactating cows, 15 dry cows, 36 heifers and 10 calves). Skin lesions were observed in 26/59 (44.1%) of the lactating cows (23 alpine gray and 3 aberdeen angus) with the age ranging between 2 and 6 years. Amongst the 26 affected animals 4 were not pregnant and the remaining cows were pregnant at 2–6 months of gestation.

**Fig. 3:**
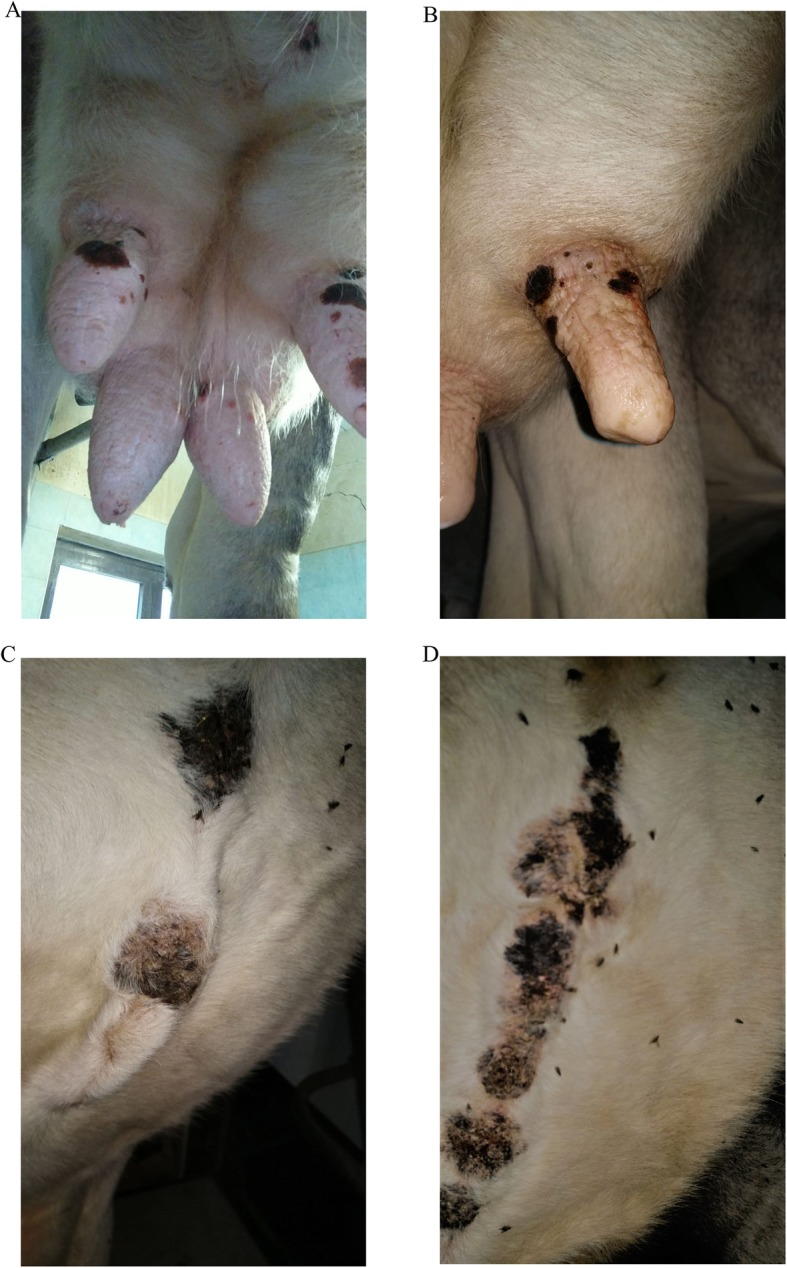
Erosive and crusted lesions on the nipples (**a** and **b**). Extensive crusted areas on the abdomen (**c** and **d**)

The affected animals were afebrile and showed lesions on the skin of the nipples, udder and ventral surface of the abdomen, near the mammary veins. The lesions varied in diameter over the stage of infection and ranged between 1 and 3 cm. Erosive skin lesions were observed, evolving into crusts (Fig. 3a and b). Along the central line of the abdomen (Fig. 3c), the lesions converged into an extensive area (Fig. 3d). All the lesions were particularly painful for the animals, chiefly the lesions located on the nipples. Pain hampered milking in the animals and the farmer opted for an early drying off of the cows. The affected animals did not recover simultaneously and clinical signs disappeared within 2 months, with the crusts falling off and the pain subsiding. The farmer reported that 1 year before the onset of the clinical form, 15 alpine grey cows were introduced in the farm from the prefecture of Bolzano, Trentino Alto Adige, Northern Italy. Amongst the 26 affected animals 2 belonged to the 15 alpine grey cows introduced in the farm.

### Sample collection

Since herpetic infection was suspected on the basis of clinical presentation, the crusts collected from the lactacting cows were subjected to molecular investigations and viral isolation. Blood samples from the same animals were collected during acute disease, defined as time 0 (T0), after 15 days (T15) and after 45 days (T45) during convalescence, to evaluate seroconversion. Moreover, 10 sera from animals apparently healthy, without skin lesions, were collected at T0, T15 and T45. Sample collection was performed upon owner’s permission.

### Viral isolation

Crust samples were processed for viral isolation. The crusts were homogenized at 10% with Dulbecco Minimal Essential Medium (D-MEM) and then centrifuged at 13,000 x g for 10 min at + 4 °C. The supernatant was treated with antibiotics for 30 min and inoculated onto 24 h-seeded MDBK cell monolayers grown in 6-well plates. The cells were incubated at 37 °C and observed daily for CPE. For haematoxylin and eosin staining and IF test, cells were grown on coverslips placed in 6-well plates.

### IF test

For the IF test, the serum of an animal recovering from the disease was used, after dilution 1:30 in PBS. Slides with BoHV-2 infected cells were fixed with cold acetone and covered with cattle serum for 30 min at 37 °C. After three washes in PBS, the slides were covered with rabbit anti-bovine IgG serum conjugated to fluoresceine isothiocyanate (FITC, Sigma Chemicals) for 30 min at 37 °C. After three washes in PBS, the slides were counterstained with Evans blue (BioMerieux) and examined under a fluorescent microscope.

### DNA extraction

Skin tissues were homogenized at 10% in D-MEM in tubes using with 2 mm-diameter steel beads vigorously vortexed with the Tissuelyser (Qiagen GmBH, Hilden, GE). Viral DNA was extracted from 200 μl of the homogenates using the QIAamp cador Pathogen Mini Kit (Qiagen S.p.A., Milan, Italy), following the manufacturer’s protocol and stored at − 80 °C until use.

### Pan-herpes PCR and nested PCR

Since herpesvirus infection was suspected on the basis of clinical presentation and CPE in cell cultures, the DNA extracts from skin tissues were subjected to a pan-herpesvirus PCR [10]. First-round PCR was performed in a final volume of 50 μl containing 5 μl of DNA extract and TaKaRa LaTaq™ kit (Takara Bio Europe S.A.S. Saint-Germain-en-Laye, France) mix consisting of: 5 μl of 10x buffer, 5 μl of MgCl_2_ (25 mM), 5 μl of Dimethyl sulfoxide (DMSO) (5.11 μM), 1 μl of forward (DFA and ILK) and reverse (KG1) primers (50 μM) [10] (Table 1), 8 μl of deoxynucleotide triphosphates (dNTPs) (2.5 mM), 0.5 μl of Takara La Taq polymerase (5 U/μl). Thermic file consisted of initial denaturation cycle at 94 °C for 1 min, followed by 45 cycles of denaturation at 94 °C for 1 min 30s, annealing at 42 °C for 1 min 30s, and extension at 68 °C for 1 min and a final extension cycle at 68 °C for 10 min.

**Table 1:** Primer/probes used in this study

Assay	Primer	Sequence 5′–3′	Amplicon size (bp)	Tm (°C)	Target	Reference
First round PCR	DFA	GAYTTYGCNAGYYTNTAYCC	315	42	DNA polymerase	[10]
	ILK	TCCTGGACAAGCAGCARNYSGCNMTNAA				
	KG1	GTCTTGCTCACCAGNTCNACNCCYTT				
Second round PCR	TGV	TGTAACTCGGTGTAYGGNTTYACNGGNGT	222		DNA polymerase	
	IYG	CACAGAGTCCGTRTCNCCRTADAT				
real time quantitative PCR (qPCR)	BoHV-2 Pol F	TGCTACGGTCACCACCATAG	105	54	DNA polymerase	this study
	BoHV-2 Pol R	TCCGGAAAGTCCATCACGAG				
	BoHV2 Pb	[FAM] CACTCGCGGTGGGTCAGCTTTGACG [BHQ1]				

Second-round PCR was performed in a final volume of 50 μl containing 5 μl of the amplicons diluted 1:100 obtained in the first PCR and TaKaRa LaTaq™ kit (Takara Bio Europe S.A.S. Saint-Germain-en-Laye, France) mix consisting of: 5 μl of 10x buffer, 5 μl of MgCl_2_ (25 mM), 1 μl of forward (TGV) and reverse (IYG) primers (50 μM) [10] (Table 1), 8 μl of deoxynucleotide triphosphates (dNTPs) (2.5 mM), 0.5 μl of Takara La Taq polymerase (5 U/μl). Thermic file consisted of initial denaturation cycle at 94 °C for 2 min, followed by 25 cycles of denaturation at 94 °C for 1 min, annealing at 42 °C for 1 min and extension at 68 °C for 1 min and a final extension cycle at 68 °C for 7 min.

The PCR products were analysed on a 1.5% agarose gel prepared in TBE buffer (0.09 M of boric acid, 0.09 M of Tris, 0.0025 M of EDTA, pH 8.3) and subjected to an electrophoresis at 50 V for 90 min. The amplification bands were visualized on a Gel Doc™ EZ (Bio-Rad Laboratories SRL, Segrate, Italy).

### Sequence analysis

The amplicons were purified and directly sequenced by Eurofins Genomics (Ebersberg, Germany). The sequences were manually edited using Geneious software version 9.1.8 (Biomatters Ltd., Auckland, New Zealand). Basic Local Alignment Search Tool (BLAST; http://www.ncbi.nlm.nih.gov) and FASTA (http://www.ebi.ac.uk/fasta33) with default values were used to find homologous hits.

### qPCR

A Real-time qPCR was designed on the sequence of the DNA-polymerase region of the isolated and characterized strain ITA/2018/468 in order to quantitate virus load. BoHV-2 DNA copy numbers were calculated on the basis of standard curves generated by 10-fold dilutions of a plasmid standard TOPO XL PCR containing a 250-nt fragment of the DNA-polymerase region of strain ITA/2018/468. A total of 10 μL of sample DNA or plasmid standard were added to the 15-μL reaction master mix (IQ Supermix; Bio-Rad Laboratories SRL, Segrate, Italy) containing 0.9 μmol/L of each primer (100 μM) and 0.1 μmol/L of probe (100 μM) (Table 1). Thermal cycling consisted of activation of iTaq DNA polymerase at 95 °C for 3 min, 45 cycles of denaturation at 95 °C for 1 min, and annealing extension at 54 °C for 30s. The specificity of the qPCR assay for BoHV-2 was evaluated using a panel of herpesviruses available in our laboratory. The panel included bovine (BoHV-1 and BoHV-4) and bubaline (BuHV-1) alphaherpesviruses.

### NGS

For DNA extraction, virus stocks were obtained from semi-purified virus particles. In brief, the cell medium of MDBK cells infected with strain ITA/2018/468 was collected at 96 h post-infection. Nucleic and cell debris were discarded by centrifugation at 17,000 x g for 3 min at 4 °C and filtration in 0.45-μm centrifugal filter [23]. Viral DNA was extracted using the DNeasy blood and tissue (QIAGEN GmBH, Germany) according to the manufacturer’s instructions.

The DNA was quantified using the Fluorometric Qubit dsDNA High Sensitivity Assay Kit (Thermo Fisher Scientific, Waltham, MA, USA). A genomic DNA library was prepared using the Nextera XT DNA Sample Prep Kit (Illumina, San Diego, CA, USA), according to the manufacturer’s protocol. A size-selection step was performed manually using Ampure XP magnetic beads (Beckman Coulter). Quality control analysis was performed on the sample library with the Agilent 2100 Bioanalyzer System (Agilent, Santa Clara, CA, USA). Library samples were normalized according to the manufacturer’s protocol and sequencing was performed with MiSeq Reagent Kit v2 by using MiSeq instrument version 2 (Illumina) to produce 2 × 250 bp paired-ended reads.

### Genome annotation and comparison

A total of 1,830,370 paired reads were obtained by NGS, with an average length of 221 bp. Quality control of reads was performed using FastQC (http://www.bioinformatics.bbsrc.ac.uk/projects/fastqc/) and sequence trimming was performed using the plugin Trim Ends in Geneious software version 9.1.8 (Biomatters Ltd., Auckland, New Zealand). NGS sequences were filtered using bovine genome (*Bos taurus*), yielding 1,603,994 reads mapped to the host genome. The unmapped reads were subjected to reference guided assembly against different complete and partial BoHV-2 gene sequences retrieved from GenBank database using the software Geneious Assembler.

### Phylogenetic analyses

Three different full-length gene targets (glycoprotein B, thymidine kinase and glycoprotein G) of ITA/2018/468 strain were aligned with cognate sequences representative of the genus *Simplexvirus* retrieved from International Committee on Taxonomy of Viruses (ICTV) (https://talk.ictvonline.org/taxonomy/) and available from GenBank database by using MAFFT algorithm [24] implemented in Geneious software version 9.1.8 (Biomatters Ltd., Auckland, New Zealand).

Phylogenetic analyses were performed with Bayesian inference by using 4 chains run for > 1 million generations [25, 26] and ModelTest software (http://evomics.org/resources/software/molecular-evolution-software/modeltest/). The identified program settings for all partitions, under the Akaike Information Criteria, included 6-character states (general time reversible model), a proportion of invariable sites, and a gamma distribution of rate variation across sites. We deposited nucleotide sequences of strain ITA/2018/468 in GenBank (accession nr MN086780–802).

### Virus neutralization

Virus neutralization (VN) was performed as described elsewhere [27]. Briefly, sera were heat-inactivated at 56 °C for 30 min and serial 2-fold dilutions starting from 1∶2 to 1:512 were mixed with 100 Tissue Culture Infection Dose (TCID_50_) of the BoHV-2 isolate in 96-well microtiter plates. Plates were held at 37 °C for 45 min, and then 20,000 MDBK cells were added to each well. After incubation for 3 days at 37 °C with 5% of CO_2_, titers were determined.

## Supplementary information

**Additional file 1: Table S1.** Nucleotide (nt) identity (express as percentage) of strain ITA/2018/468 with 23 different gene targets of reference BoHV-2 strains retrieved from GenBank database.

## Data Availability

The data that support the findings of this study are available from the corresponding author upon reasonable request. Nucleotide sequences of strain ITA/2018/468 were deposited in GenBank (accession nr MN086780–802).

## Abbreviations

BoHV: Bovine alphaherpesvirus
LSD: Lumpy skin disease
PSLD: Pseudo-Lumpy Skin Disease
BHM: Bovine Herpetic Mammillitis
MDBK: Madin Darby Bovine Kidney
nt: nucleotide
D-MEM: Dulbecco Minimal Essential Medium
IF: Immunofluorescence
CPE: Cytopathic effect
BLAST: Basic Local Alignment Search Tool
NGS: Next Generation Sequencing
LepHV-4: Leporid alphaherpesvirus 4
MacHV-2: Macropodid alphaherpesvirus 2
ICTV: International Committee on Taxonomy of Viruses
TCID_50_: Tissue Culture Infection Dose
