# Supplementary material for: Isolation and characterization of bovine alphaherpesvirus 2 strain from an outbreak of bovine herpetic mammillitis in a dairy farm
Source: BMC Vet Res. 2020 Mar 30;16:103. doi: 10.1186/s12917-020-02325-3 (PMC7106810; doi:10.1186/s12917-020-02325-3)
Supplement: Supplementary file 1 — Additional file 1: Table S1. Nucleotide (nt) identity (express as percentage) of strain ITA/2018/468 with 23 different gene targets of reference BoHV-2 strains retrieved from GenBank database. [file 12917_2020_2325_MOESM1_ESM.docx]

| ITA/2018/468 | AF387490 %nt | AY357735 %nt | AY357736 %nt | AY027921 %nt | AF375976 %nt | AF439863 %nt | D00537 %nt | AY098932 %nt | AY064482 %nt | AY046323 %nt | AF409104 %nt | AF410478 %nt | M21628 %nt | AF383175 %nt | AF181249 %nt | AF486631 %nt | AF326961 %nt | AF383176 %nt | AY033933 %nt | AY120887 %nt | AF515806 %nt | AY101384 %nt | AY099481 %nt | AF372518 %nt | AF383177 %nt | AF161021 %nt |
| --- | --- | --- | --- | --- | --- | --- | --- | --- | --- | --- | --- | --- | --- | --- | --- | --- | --- | --- | --- | --- | --- | --- | --- | --- | --- | --- |
| tegument protein | **99.9** | **-** | **-** | **-** | **-** | **-** | **-** | **-** | **-** | **-** | **-** | **-** | **-** | **-** | **-** | **-** | **-** | **-** | **-** | **-** | **-** | **-** | **-** | **-** | **-** | **-** |
| capsid protein | **-** | **100** | **-** | **-** | **-** | **-** | **-** | **-** | **-** | **-** | **-** | **-** | **-** | **-** | **-** | **-** | **-** | **-** | **-** | **-** | **-** | **-** | **-** | **-** | **-** | **-** |
| major capsid protein | **-** | **-** | **99.8** | **-** | **-** | **-** | **-** | **-** | **-** | **-** | **-** | **-** | **-** | **-** | **-** | **-** | **-** | **-** | **-** | **-** | **-** | **-** | **-** | **-** | **-** | **-** |
| integral membrane protein | **-** | **-** | **-** | **99.8** | **-** | **-** | **-** | **-** | **-** | **-** | **-** | **-** | **-** | **-** | **-** | **-** | **-** | **-** | **-** | **-** | **-** | **-** | **-** | **-** | **-** | **-** |
| glycoprotein H | **-** | **-** | **-** | **-** | **99.2** | **-** | **-** | **-** | **-** | **-** | **-** | **-** | **-** | **-** | **-** | **-** | **-** | **-** | **-** | **-** | **-** | **-** | **-** | **-** | **-** | **-** |
| thymidine kinase | **-** | **-** | **-** | **-** | **-** | **99.8** | **99.9** | **-** | **-** | **-** | **-** | **-** | **-** | **-** | **-** | **-** | **-** | **-** | **-** | **-** | **-** | **-** | **-** | **-** | **-** | **-** |
| non-glycosylated membrane-associated protein | **-** | **-** | **-** | **-** | **-** | **-** | **-** | **99.5** | **99.7** | **100** | **-** | **-** | **-** | **-** | **-** | **-** | **-** | **-** | **-** | **-** | **-** | **-** | **-** | **-** | **-** | **-** |
| capsid associated tegument | **-** | **-** | **-** | **-** | **-** | **-** | **-** | **-** | **-** | **-** | **99.7** | **-** | **-** | **-** | **-** | **-** | **-** | **-** | **-** | **-** | **-** | **-** | **-** | **-** | **-** | **-** |
| capsid maturation protease | **-** | **-** | **-** | **-** | **-** | **-** | **-** | **-** | **-** | **-** | **-** | **99.9** | **-** | **-** | **-** | **-** | **-** | **-** | **-** | **-** | **-** | **-** | **-** | **-** | **-** | **-** |
| glycoprotein B | **-** | **-** | **-** | **-** | **-** | **-** | **-** | **-** | **-** | **-** | **-** | **-** | **99.8** | **-** | **-** | **-** | **-** | **-** | **-** | **-** | **-** | **-** | **-** | **-** | **-** | **-** |
| nuclear phosphoprotein | **-** | **-** | **-** | **-** | **-** | **-** | **-** | **-** | **-** | **-** | **-** | **-** | **-** | **99.8** | **-** | **-** | **-** | **-** | **-** | **-** | **-** | **-** | **-** | **-** | **-** | **-** |
| DNA-dependent DNA polymerase | **-** | **-** | **-** | **-** | **-** | **-** | **-** | **-** | **-** | **-** | **-** | **-** | **-** | **-** | **99.9** | **-** | **-** | **-** | **-** | **-** | **-** | **-** | **-** | **-** | **-** | **-** |
| terminase | **-** | **-** | **-** | **-** | **-** | **-** | **-** | **-** | **-** | **-** | **-** | **-** | **-** | **-** | **-** | **100** | **-** | **-** | **-** | **-** | **-** | **-** | **-** | **-** | **-** | **-** |
| ribonucleotide reductase protein | **-** | **-** | **-** | **-** | **-** | **-** | **-** | **-** | **-** | **-** | **-** | **-** | **-** | **-** | **-** | **-** | **99.7** | **-** | **-** | **-** | **-** | **-** | **-** | **-** | **-** | **-** |
| nuclear protein | **-** | **-** | **-** | **-** | **-** | **-** | **-** | **-** | **-** | **-** | **-** | **-** | **-** | **-** | **-** | **-** | **-** | **99.8** | **-** | **-** | **-** | **-** | **-** | **-** | **-** | **-** |
| virion host shutoff protein | **-** | **-** | **-** | **-** | **-** | **-** | **-** | **-** | **-** | **-** | **-** | **-** | **-** | **-** | **-** | **-** | **-** | **-** | **100** | **-** | **-** | **-** | **-** | **-** | **-** | **-** |
| DNA polymerase processivity factor | **-** | **-** | **-** | **-** | **-** | **-** | **-** | **-** | **-** | **-** | **-** | **-** | **-** | **-** | **-** | **-** | **-** | **-** | **-** | **98.9** | **-** | **-** | **-** | **-** | **-** | **-** |
| membrane protein | **-** | **-** | **-** | **-** | **-** | **-** | **-** | **-** | **-** | **-** | **-** | **-** | **-** | **-** | **-** | **-** | **-** | **-** | **-** | **-** | **99.9** | **-** | **-** | **-** | **-** | **-** |
| glycoprotein C | **-** | **-** | **-** | **-** | **-** | **-** | **-** | **-** | **-** | **-** | **-** | **-** | **-** | **-** | **-** | **-** | **-** | **-** | **-** | **-** | **-** | **98.8** | **-** | **-** | **-** | **-** |
| tegument envelope protein | **-** | **-** | **-** | **-** | **-** | **-** | **-** | **-** | **-** | **-** | **-** | **-** | **-** | **-** | **-** | **-** | **-** | **-** | **-** | **-** | **-** | **-** | **100** | **-** | **-** | **-** |
| helicase primase | **-** | **-** | **-** | **-** | **-** | **-** | **-** | **-** | **-** | **-** | **-** | **-** | **-** | **-** | **-** | **-** | **-** | **-** | **-** | **-** | **-** | **-** | **-** | **99.6** | **-** | **-** |
| nuclear matrix-associated protein | **-** | **-** | **-** | **-** | **-** | **-** | **-** | **-** | **-** | **-** | **-** | **-** | **-** | **-** | **-** | **-** | **-** | **-** | **-** | **-** | **-** | **-** | **-** | **-** | **99.5** | **-** |
| glycoprotein G | **-** | **-** | **-** | **-** | **-** | **-** | **-** | **-** | **-** | **-** | **-** | **-** | **-** | **-** | **-** | **-** | **-** | **-** | **-** | **-** | **-** | **-** | **-** | **-** | **-** | **98.9** |

Supplementary Table 1: nucleotide (nt) identity (express as percentage) of strain ITA/2018/468 with 23 different gene targets of reference BoHV-2 strains retrieved from GenBank database.
